# Supplementary material for: All-at-once RNA folding with 3D motif prediction framed by evolutionary information
Source: Nat Methods. 2025 Oct 3;22(10):2094–106. doi: 10.1038/s41592-025-02833-w (PMC12510878; doi:10.1038/s41592-025-02833-w)
Supplement: Supplementary file 3 — Source code to run CaCoFold-R3D. Data and results. [file 41592_2025_2833_MOESM3_ESM.gz › supplemental_material/Figures/Figure_3.pdf]

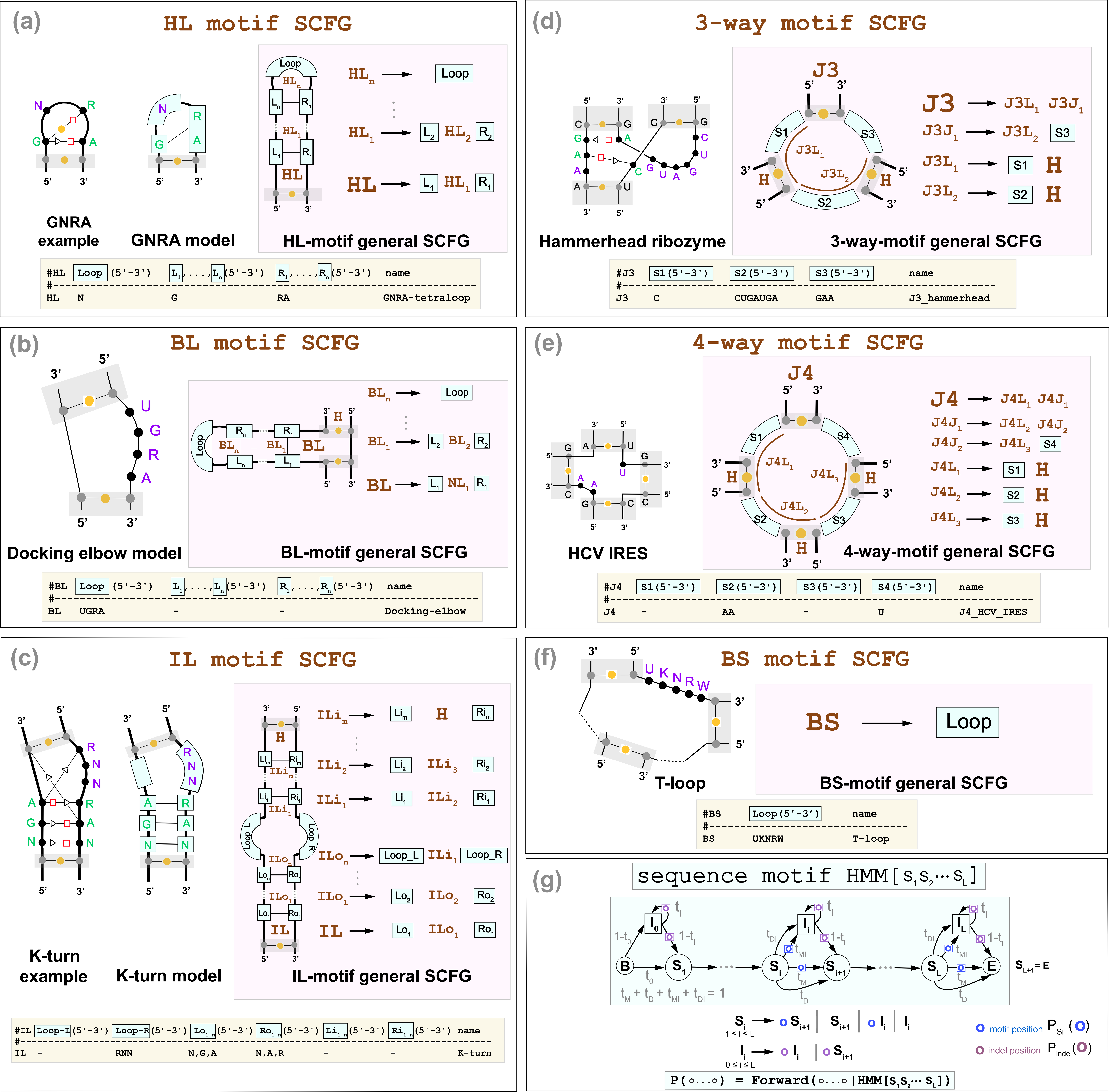

(a) HL motif SCFG
GNRA example
GNRA model
HL-motif general SCFG
HLn → Loop
HL1 → L2 HL2 R2
HL → L1 HL1 R1
#HL Loop(5'-3') L1,...,Ln(5'-3') R1,...,Rn(5'-3') name
HL N G RA GNRA-tetraloop
(b) BL motif SCFG
Docking elbow model
BL-motif general SCFG
BLn → Loop
BL1 → L2 BL2 R2
BL → L1 NL1 R1
#BL Loop(5'-3') L1,...,Ln(5'-3') R1,...,Rn(5'-3') name
BL UGRA - - Docking-elbow
(c) IL motif SCFG
K-turn example
K-turn model
IL-motif general SCFG
ILim → Lim H Rim
ILi2 → Li2 ILi3 Ri2
ILi1 → Li1 ILi2 Ri1
ILon → Loop_L ILi1 Loop_R
ILo1 → Lo2 ILo2 Ro2
IL → Lo1 ILo1 Ro1
#IL Loop-L(5'-3') Loop-R(5'-3') Lo1-n(5'-3') Ro1-n(5'-3') Li1-n(5'-3') Ri1-n(5'-3') name
IL - RNN N,G,A N,A,R - - K-turn
(d) 3-way motif SCFG
Hammerhead ribozyme
3-way-motif general SCFG
J3 → J3L1 J3J1
J3J1 → J3L2 S3
J3L1 → S1 H
J3L2 → S2 H
#J3 S1(5'-3') S2(5'-3') S3(5'-3') name
J3 C CUGAUGA GAA J3_hammerhead
(e) 4-way motif SCFG
HCV IRES
4-way-motif general SCFG
J4 → J4L1 J4J1
J4J1 → J4L2 J4J2
J4J2 → J4L3 S4
J4L1 → S1 H
J4L2 → S2 H
J4L3 → S3 H
#J4 S1(5'-3') S2(5'-3') S3(5'-3') S4(5'-3') name
J4 - AA - U J4_HCV_IRES
(f) BS motif SCFG
T-loop
BS-motif general SCFG
BS → Loop
#BS Loop(5'-3') name
BS UKNRW T-loop
(g) sequence motif HMM[S1S2···SL]
tM + tD + tMI + tDI = 1
SL+1 = E
Si → o Si+1 | Si+1 | o Ii | Ii (1 ≤ i ≤ L)
Ii → o Ii | o Si+1 (0 ≤ i ≤ L)
motif position PSi(O)
indel position Pindel(O)
P(o...o) = Forward(o...o | HMM[S1S2··· SL])
